# Disinfection of Railway Coaches

**Published:** 1899-11

**Authors:** 


### Disinfection of Railway Coaches.

The danger of infection of railway coaches is so obvious from expectorations on the floor and the indiscriminate character of the passengers carried that some measures would be naturally suggested to any one interested in sanitary matters. For this disinfection, Warner here reports results of experiments made with formaldehyde gas in two railway cars. Test cultures were placed within the cars, the windows and doors shut, the other openings stuffed with waste, and the generator placed on the platform, the gas being conducted by a tube through the keyhole. The day was unfavorable, there being a high wind, increasing the ventilation, but in one experiment eight out of thirteen cultures gave no growth, and in the other, six out of seventeen only gave growth. Considering the methods, the results are rather significant, and he thinks that the needs could be met by requiring the regular disinfection of the cars by the railway companies.—*Virginia Medical Semi-Monthly.*

